# Cerebral microdialysis in clinical studies of drugs: pharmacokinetic applications

**DOI:** 10.1007/s10928-013-9306-4

**Published:** 2013-03-07

**Authors:** Richard J. Shannon, Keri L. H. Carpenter, Mathew R. Guilfoyle, Adel Helmy, Peter J. Hutchinson

**Affiliations:** 1 Division of Neurosurgery, Department of Clinical Neurosciences, University of Cambridge, Box 167, Addenbrooke’s Hospital, Cambridge, CB2 0QQ UK; 2 Wolfson Brain Imaging Centre, Department of Clinical Neurosciences, University of Cambridge, Box 65, Addenbrooke’s Hospital, Cambridge, CB2 0QQ UK

**Keywords:** Brain, Microdialysis, Pharmacokinetics, Human

## Abstract

The ability to deliver drug molecules effectively across the blood–brain barrier into the brain is important in the development of central nervous system (CNS) therapies. Cerebral microdialysis is the only existing technique for sampling molecules from the brain extracellular fluid (ECF; also termed interstitial fluid), the compartment to which the astrocytes and neurones are directly exposed. Plasma levels of drugs are often poor predictors of CNS activity. While cerebrospinal fluid (CSF) levels of drugs are often used as evidence of delivery of drug to brain, the CSF is a different compartment to the ECF. The continuous nature of microdialysis sampling of the ECF is ideal for pharmacokinetic (PK) studies, and can give valuable PK information of variations with time in drug concentrations of brain ECF versus plasma. The microdialysis technique needs careful calibration for relative recovery (extraction efficiency) of the drug if absolute quantification is required. Besides the drug, other molecules can be analysed in the microdialysates for information on downstream targets and/or energy metabolism in the brain. Cerebral microdialysis is an invasive technique, so is only useable in patients requiring neurocritical care, neurosurgery or brain biopsy. Application of results to wider patient populations, and to those with different pathologies or degrees of pathology, obviously demands caution. Nevertheless, microdialysis data can provide valuable guidelines for designing CNS therapies, and play an important role in small phase II clinical trials. In this review, we focus on the role of cerebral microdialysis in recent clinical studies of antimicrobial agents, drugs for tumour therapy, neuroprotective agents and anticonvulsants.

## Introduction

Our knowledge and understanding of the central nervous system (CNS) disorders has increased greatly in the last two decades, along with rapidly advancing technology. However, these advances have not been matched by an increase in the number of new pharmacological agents to treat and prevent neurological and neuropsychiatric disorders. The development of new CNS drugs is fraught with difficulty. A recent survey reports that as little as 8 % of CNS drug candidates ever become available for clinical use, compared with 15 % of other drugs [1]. The report also suggests that trial failures tend to occur later in the clinical development process, when costs are highest. Reasons for failures include inadequate clinical efficacy, inadequate clinical safety (e.g. harmful side effects), and lack of detailed and accurate information about how the drug enters and functions in the brain. In order to translate our knowledge and understanding into clinical applications and novel therapies, more detailed early clinical studies need to be carried out to select drug candidates that are likely to be successful in clinical trials. Especially important are pharmacokinetic studies that help us to gain a better understanding of drug bioavailability in, and elimination from, the human brain.

An earlier review by Helmy et al. [2] gives a thorough account of the microdialysis method and highlights its potential role in the development and clinical assessment of drugs. Alavijeh and Palmer [3] have recently reviewed the pharmacokinetic and pharmacodynamic properties of neuroactive compounds, concentrating on cerebral microdialysis studies using animals. An older review by de la Peña et al. [4] gives an overview of the use of microdialysis in peripheral tissues. In the present review, we focus on recent drug studies using microdialysis to sample the extracellular fluid of the human brain.

### The compartments of the brain

Developing improved treatments for CNS disorders such as epilepsy, traumatic brain injury (TBI), stroke, brain tumours, Parkinson’s disease and Alzheimer’s disease requires more accurate and detailed data about human neuro-pharmacokinetics and neurochemistry. Disorders of the brain involve biochemical mediators, so the monitoring and manipulation of brain chemistry is essential for developing and evaluating new treatments. Neuroactive drugs are likely to interact with endogenous pathways in the brain involving such species as neurotransmitters, amino acids, reactive oxygen species, membrane transporters and enzymes. An added complexity of treating CNS disorders is the strict compartmentalisation of the brain interstitium from the blood circulation by the blood–brain barrier (BBB) (see section entitled “The blood-brain barrier”).

**Fig. 1:**
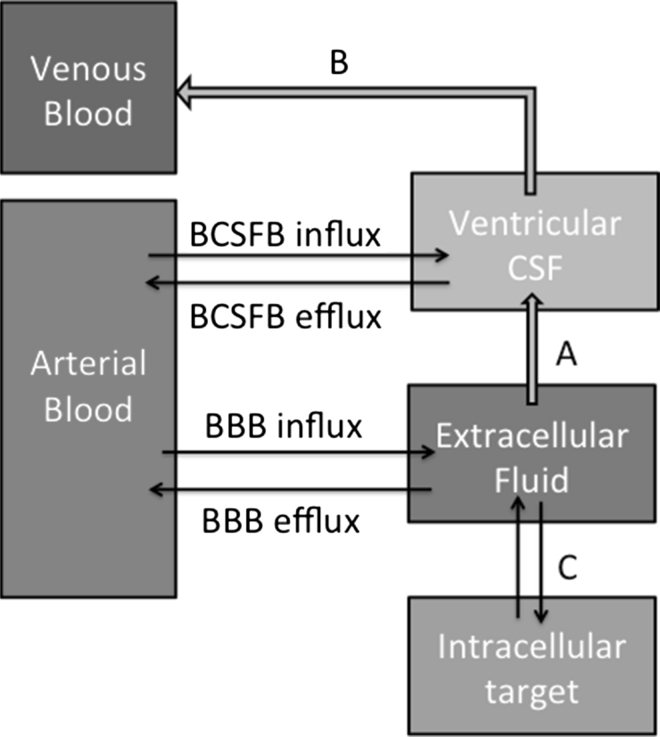
Extracellular fluid is the key compartment for the action of a neuroactive compound. The diagram is adapted from Alavijeh et al. [7] and Shen et al. [8]. BBB is Blood–brain barrier. BCSFB is blood–cerebrospinal fluid barrier. BCSFB influx and efflux is via the choroid plexus, which is also the major source of CSF. *A* Bulk flow of ECF to CSF with no barrier. *B* CSF fluid (and solutes) are absorbed into venous blood via the arachnoid villi. *C* Uptake of drug into cells is either passive or transporter driven, similarly for efflux from cells. Biological effect is usually due to interaction of drug with a membrane receptor or an intracellular target

In order to interact with molecules or receptors inside the brain, a drug must first enter the brain and be sustainable at a pharmacologically relevant concentration at the target site. Small, lipophilic molecules can diffuse through the BBB to get into the brain. Otherwise, a drug must either be transported by a membrane transporter or gain access through compromised BBB [5, 6]. Once inside the brain, drug molecules can be unbound (free) in the extracellular fluid (ECF), or taken up into cells or bound to membranes or extracellular matrix. Drugs can also be transported back out of the brain by an efflux transporter. The proportion of free drug that remains in the ECF can gain access to glial cells and neurons, or bind to membrane transporters, carrying out its pharmacological function at the target site (Fig. 1) [7, 8]. Therefore, developing neuroactive drugs requires an understanding of the pharmacokinetics of the drug in the brain as well as in blood. Being able to measure the amount of free drug in brain ECF is essential to assess whether a putative neuroactive drug is likely to work.

The clearance of a drug from the brain is also complex. The ECF drains (passively) into the cerebrospinal fluid (CSF); there is no barrier to this transport [9]. Chemicals in the ECF may find their way into the CSF by this route or they may be actively transported back into the blood, across the BBB. If the drug is actively transported out of the ECF, it may be difficult to maintain a pharmacologically relevant concentration at the target site. For this reason, information about how the drug is cleared from the brain should also be evaluated in early clinical studies.

Cerebral microdialysis is the only technique that enables us to directly sample molecules from the brain ECF. It is therefore the gold standard technique for evaluating CNS drug pharmacokinetics in vivo during early drug development [10].

### Cerebral microdialysis

**Fig. 2:**
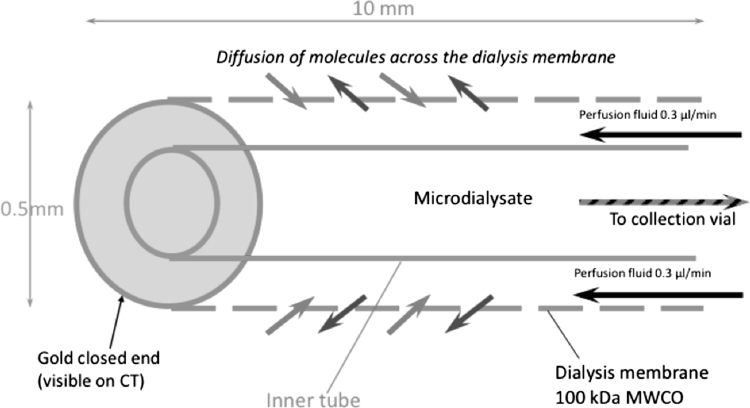
Schematic of the microdialysis catheter tip. Substances in the extracellular fluid outside the catheter tip are able to diffuse across the microdialysis membrane to be collected for analysis

Microdialysis enables the chemistry of ECF in body tissues to be monitored. The technique has been used in patients with severe brain conditions such as TBI, subarachnoid haemorrhage, brain tumours and epilepsy. A microdialysis catheter tip is implanted into the cerebral parenchyma (brain tissue). The catheter tip consists of two concentric tubes where the outer wall is a semi-permeable microdialysis membrane. Perfusion fluid (a solution of 147 mM NaCl, 2.7 mM KCl, 1.2 mM CaCl_2_, 0.85 mM MgCl_2_ in water) is pumped through the space between the dialysis membrane and the inner tube at a low flow rate, typically 0.3 μl/min. The microdialysis membrane allows molecules to diffuse in both directions between the brain ECF and the perfusion fluid in the catheter. The end of the catheter is closed so that the perfusion fluid returns back up the central tube of the catheter to be collected in a vial (Fig. 2). The catheter enables sampling of the molecules from the ECF of the tissue into which it has been implanted.

Substances of interest in brain ECF include markers of brain metabolism (e.g. glucose, lactate, pyruvate), neurotransmitters (e.g. glutamic acid, aspartic acid, GABA), markers of membrane damage (e.g. glycerol) and inflammation (e.g. cytokines), as well as drugs.

**Fig. 3:**
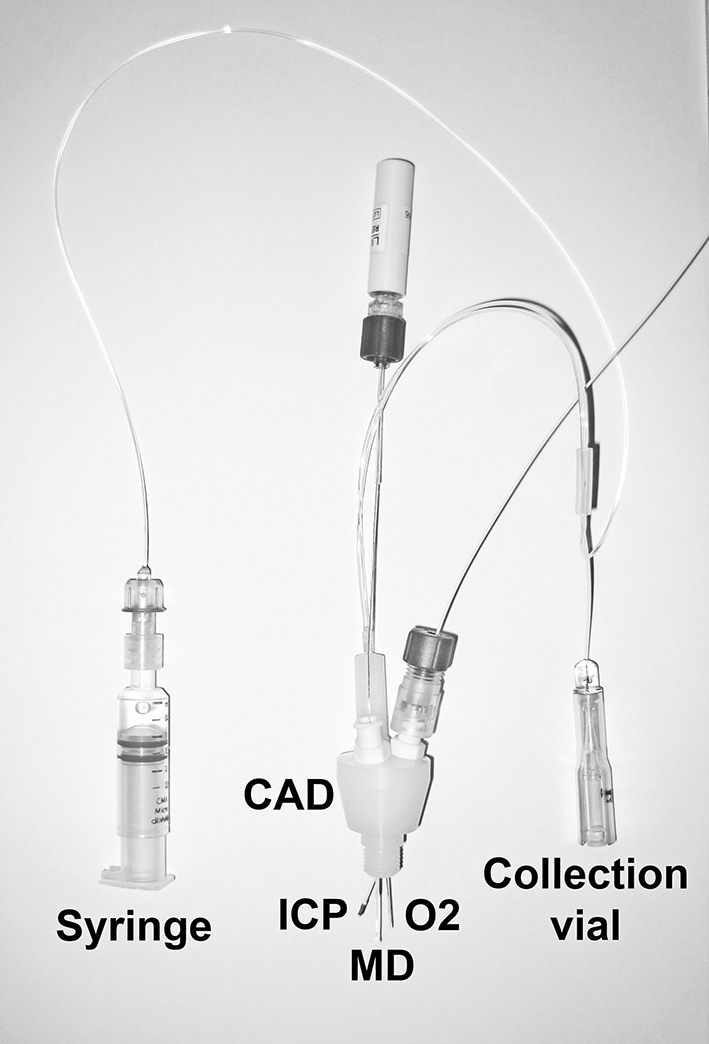
A triple lumen cranial access device (CAD) is inserted into the skull, to provide access into the brain for the microdialysis catheter (MD) and for sensors measuring intracranial pressure (ICP) and brain tissue oxygen concentration (O_2_). A pump (not shown) drives the syringe that delivers perfusion fluid into the microdialysis catheter, and the microdialysate emerges from the brain into a collection vial. The vial is changed hourly by a nurse and analysed at the bedside on a clinical microdialysis analyser (ISCUS or CMA600, for glucose, lactate, pyruvate, glutamate and glycerol) and in the laboratory for other analytes (e.g. drugs) as desired. Image copyright K.L.H. Carpenter and reproduced here with her permission

There are many examples where microdialysis has been used in animal studies to help elucidate the neuro-pharmacokinetics of certain drugs [3]. Excellent data can be gathered from regular blood sampling and cerebral microdialysis, and conclusions drawn about the interplay between blood and brain. These studies are used to predict therapeutic doses and dosing intervals for human clinical trials. However, there may be significant differences between how the drug behaves in an animal model and in humans. For example, there may be different substrate-specific transporters operating at the BBB (see section entitled “Genetic polymorphism and BBB transporters in patients”). The most relevant pharmacokinetic data come from human studies. However, cerebral microdialysis is an invasive technique that cannot be applied to healthy human volunteers. Cerebral microdialysis studies have been carried out on patients undergoing biopsy or tumour resection, surgery for severe epilepsy, or undergoing neurocritical care after severe brain injury (e.g. head injury and certain types of stroke) where microdialysis is part of multimodality monitoring. Microdialysis catheters are inserted into the brain either via a craniotomy or via a burr-hole. A triple-lumen cranial access device (Technicam, Newton Abbot, UK) can be used to insert the microdialysis catheter alongside probes for measuring intracranial pressure and brain tissue oxygen, all through the same burr hole, for multimodality monitoring (Fig. 3) [11]. Sometimes two microdialysis catheters—for example, one in abnormal brain (usually via a craniotomy) in the vicinity of a focal lesion, and one in brain distant from a focal lesion (usually via a cranial access device through a burr hole)—are deployed within the same patient.

### The blood–brain barrier

The BBB is a specialised neurovascular unit formed by neurons, astrocytes, pericytes, basal lamina, and endothelial cells. In contrast to the relatively permeable fenestrated endothelium of the peripheral vasculature, the endothelial cells of brain microvessels are linked by apical tight junctions and form a continuous barrier that prohibits paracellular diffusion of all but small (<400 Da) lipophilic molecules. Exchange of molecules between blood and brain interstitum is thus highly restricted and regulated by multiple families of solute transporters for small molecules (e.g. glucose and amino acids) and endo- and trans-cytosis systems for larger proteins [12, 13]. Furthermore, the BBB possesses highly effective efflux transporters to remove toxins and drugs from the CNS (Schwab et al. [12]). In combination, these mechanisms present significant challenges to developing therapeutic agents that efficiently penetrate and are sustained in the brain at pharmacologically relevant concentrations. Accordingly, drugs with CNS actions are generally highly lipophilic (e.g. anaesthetic agents), are mimetics of natural substrates to BBB transporters, or are dependent on BBB disruption to enter the CNS. Fortuitously from this perspective, increased BBB permeability is a common feature of many CNS pathologies (e.g. meningitis, primary and secondary tumours, intracerebral haemorrhage, and traumatic injury). If the BBB is not significantly affected by a disease process, therapeutic strategies have been developed to temporarily open the BBB by intravenous infusion of hypertonic agents (e.g. mannitol) prior to drug delivery [13] or to co-administer agents that block efflux transporters [14].

## Cerebral microdialysis in clinical studies of drugs

Microdialysis enables measurement of drug concentration in brain ECF, on a continuous basis. Microdialysis can thus demonstrate whether a drug in question, at an appropriate dosage, can cross the BBB at sufficient concentration. Resources can then be channelled to drug candidates that have the best chance of showing efficacy in larger-scale clinical trials.

Data from a small clinical study involving only a few patients in a neurocritical care unit may be sufficient to show whether a particular drug candidate might be worth pursuing or not. A microdialysis study by Hutchinson et al. [15] investigated the effect of the potentially neuroprotective drug chlormethiazole on neurochemistry in five TBI patients. This drug proved to be undetectable in brain microdialysates, suggesting an adequate concentration of the drug did not reach the target site to exert its mechanism of action. Interestingly, in a phase III clinical trial chlormethiazole did not improve the outcome in patients with major ischemic stroke [16]. This expensive, time-consuming and unsuccessful phase III trial could potentially have been avoided by first carrying out an in vivo microdialysis study [10].

Cerebral microdialysis is not only useful for assessing drug penetration into the brain, but is also used to monitor the effect of neuroactive drugs on brain chemistry. Monitoring endogenous compounds such as neurotransmitters and metabolic markers can provide evidence of whether a drug is affecting its target in the desired manner. It can also be used to help assess the clinical safety of the drug, e.g. whether there are any adverse changes in microdialysate levels of glucose, lactate, pyruvate, glutamate and glycerol.

The following section highlights a number of studies where cerebral microdialysis has been used to extract pharmacokinetic parameters from small clinical studies. The examples are arranged by therapeutic area: antibacterial agents, tumour therapy, neuroprotective agents and anticonvulsant drugs.

### Antibacterial agents

CNS infections such as bacterial meningitis are routinely treated with β-lactam antibiotics, such as the carbapenems. Antibiotics for treating CNS infections must be able to maintain a minimum inhibitory concentration (MIC) in the brain. The MIC of a drug is the concentration required to kill a particular strain of bacteria and is measured in vitro. These antibiotics, like any neuroactive drug, must not have a neurotoxic effect at therapeutic concentrations. Some of the carbapenem antibiotics, such as imipenem, can cause an increase in the frequency of seizures if over-dosed [17]. Others, such as meropenem, are less neurotoxic and therefore safe to use for the treatment of bacterial meningitis. In the following two examples, cerebral microdialysis has been used to evaluate the actual concentration of drug in the brain.

In a study by Dahyot-Fizelier et al. [18] an intravenous infusion containing meropenem was given to two patients being treated for acute brain injury in the neurocritical care unit (NCCU). Microdialysis sampling of brain ECF and other routine monitoring used in the NCCU was carried out, and blood samples were taken. In another study by Poeppl et al. [19], an intravenous infusion containing the new broad-spectrum antibiotic, doripenem, was given to five neuro-intensive care patients, and they were similarly monitored.

**Fig. 4:**
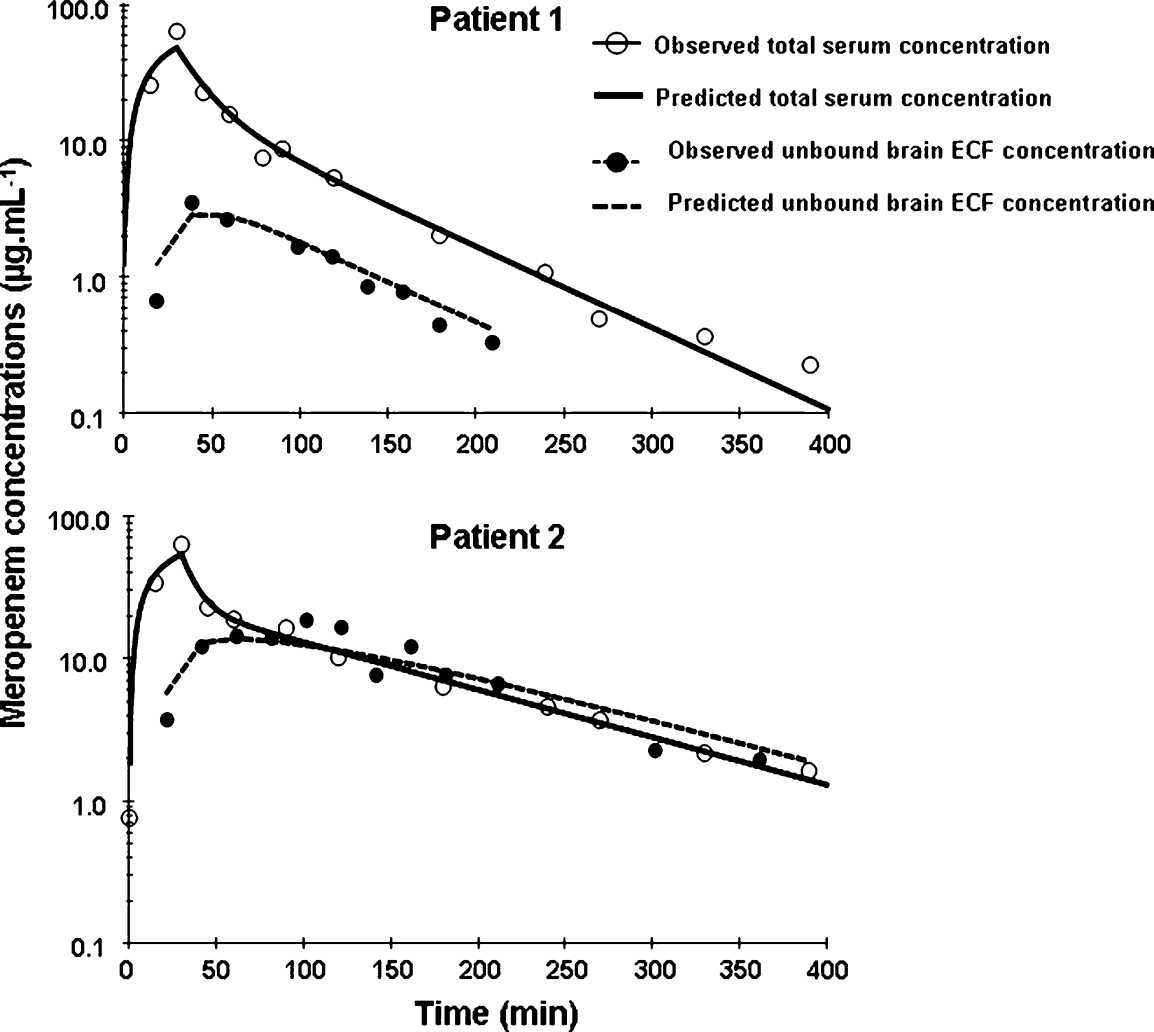
Pharmacokinetics of meropenem in serum and brain ECF, from a study by Dahyot-Fizelier et al. [18]. Individual concentrations of meropenem in serum (*white circle*), and in brain extracellular fluid (ECF) measured using microdialysis (*black circle*), plotted versus time in two critical-care patients (acute brain injury) after a 30-min intravenous infusion of 1 g of meropenem administered every 8 h during a multiple-dosing regimen. The *solid line* represents the predicted concentrations in serum, and the *dashed line* represents the predicted concentrations in brain. Copyright: American Society for Microbiology [Antimicrobial Agents and Chemotherapy, 54: 3502–3504, doi:10.1128/AAC.01725-09] and reproduced with permission

Dahyot-Fizelier et al. [18] found that the brain ECF concentrations of meropenem were lower than the blood serum concentrations. The ratio of drug in brain ECF to drug in serum was calculated from the area under the concentration–time curves (AUC) and found to be 0.73 and 0.14 for the two patients. The time course of meropenem concentration in serum and brain ECF (Fig. 4) showed delayed distribution in brain ECF from the blood. The authors developed a pharmacokinetic model to fit their experimental data, and thereby estimated various pharmacokinetic parameters for blood and brain. They concluded that the brain ECF concentration was lower than the serum concentration due to active efflux transport systems at the BBB. It is interesting to compare the pharmacokinetic results of this study with an older study by Nau et al. [20] who measured the meropenem concentration in CSF and serum from ten patients with an external ventricular drain. These patients had occlusive hydrocephalus resulting from cerebrovascular causes. Nau et al. [20] found a CSF-to-serum ratio of 0.047 ± 0.022, about one-tenth of the ECF-to-serum ratio found by Dahyot-Fizelier et al. [18] in the microdialysis study. This might be due to a difference in penetration of the two brain compartments (ECF and CSF), and/or to differences in pathology of the patients being studied.

The second microdialysis study example of an antibacterial agent was by Poeppl et al. [19], who found AUC values for doripenem in brain ECF were much lower than those for blood. The brain-to-serum AUC ratio for one of the head-injured patients in this study was 0.17, which is the same order of magnitude as for the meropenem patients. However, the AUC ratio for the remaining four patients was 0.01, indicating that very little drug appeared to cross the BBB. The authors found that the concentration of doripenem was so low that the drug would not reach the required time above the MIC for many strains of bacteria. The authors speculated that doripenem might not be a substrate for a BBB influx transporter, or that inhibition of, or genetic polymorphism in a particular transporter affects the drug concentration in brain ECF. They also suggested that the high AUC ratio seen in one patient could be due to local impairment of BBB integrity in that individual.

These two studies highlight one of the complications of carrying out pharmacokinetic studies of a drug on a small number of head injury patients: the patients are a heterogeneous group of individuals. They will inevitably have genetic differences and will have suffered varying degrees of brain injury, leading to variable loss of BBB integrity. An abnormal BBB is likely to be more permeable than normal BBB and therefore facilitate drug entry into the brain [21]. The drug may be a substrate for a number of receptors or transporters, and interact with many molecular species present in the brain. Aberrant states of brain chemistry often prevail after TBI (and vary with time, as well as within- and between-patient), evidenced by elevations in lactate, lactate/pyruvate ratio, glutamate and glycerol [74, 75], suggesting hypoxia and/or mitochondrial dysfunction, excitotoxicity and cell membrane breakdown, all of which could potentially affect the pharmacokinetics and pharmacodynamics of a drug in the brain. Also, we do not know which factors affect drug influx and efflux for a particular drug and patient. The patients may have numerous clinical features that will influence drug distribution in the blood and within brain (e.g. sites close to and distant from focal lesions). The above considerations may limit the potential to extrapolate pharmacokinetic findings from critically injured patients to a different (non-TBI) patient group.

Given the above limitations, the above two studies (meropenem and doripenem) are good examples of using cerebral microdialysis in human patients to extract basic pharmacokinetic parameters such as AUC, maximum concentration of drug (C_max_), time taken to reach C_max_, elimination rate constants and half-life of drug in brain, in parallel with blood pharmacokinetics. Pharmacokinetic analysis is possible because of the quality and quantity of data that can be accessed by microdialysis, which is a continuous sampling technique. The CSF can be taken from patients, though this fluid is less relevant for the action of a neuroactive drug than the ECF. We postulate that the brain extracellular space (which can be accessed by microdialysis) is a more relevant compartment than CSF, in the context of biological activity, since the ECF is directly in contact with neurons and astrocytes, whereas CSF is not. CSF samples can be taken from sedated patients on the neuro-intensive care unit using an external ventricular drain from a brain ventricle, or CSF can be taken by lumbar puncture from the spinal subarachnoid space. In the latter case, sampling would be much less frequent than microdialysis sampling. Furthermore, Shore et al. [22] have demonstrated that the volume and frequency of CSF sampling has an effect on the measured concentration of several cytokines in the CSF. This would suggest that CSF is acting as a ‘sump’ or excretion mechanism from the brain rather than accurately reflecting brain biology and emphasises the point that the CSF and ECF compartments are distinct entities [23].

As a comparison with the study by Poeppl et al., Margentis et al. [24] reported the CSF concentration of doripenem in five neurosurgical patients during implantation of an intrathecal baclofen infusion pump to treat spasticity. The CSF samples were taken from the spinal subarachnoid space at different times after intravenous infusion of doripenem, given prophylactically prior to the operation. None of the patients had active neurological diseases or infections, and the authors regarded the BBB as intact. They were unable to carry out pharmacokinetic calculations because only one sample was taken from each patient. However, the study did suggest that doripenem penetrated the intact BBB of these patients.

Other clinical studies of antibacterial agents by cerebral microdialysis include vancomycin in TBI patients (Caricato et al. [25]), rifampicin in brain tumour patients (Mindermann et al. [26]), and cefotaxime in a TBI patient (Frasca et al. [27]). Microdialysis can thus provide important evidence as to whether sufficient concentrations can be attained in brain to kill bacteria, although with the caveat that BBB permeability may vary considerably between patients. In their recent review, Notkina et al. [28] have remarked that treatment of cerebral infections is still a challenge and that some of the dosages of antibacterial drugs may be inadequate for more resistant bacteria despite seemingly effective plasma concentrations. Cerebral microdialysis studies with pharmacokinetic/pharmacodynamic modelling allow prediction of effectiveness of altered drug regimens and can thus form the basis of more accurate dosage for patients with CNS infections [28].

The two microdialysis studies of the antimicrobial agents meropenem and doripenem demonstrate another important point: the different approaches to calibrating the relative recovery (also termed extraction efficiency) of the microdialysis catheters. Dahyot-Fizelier et al. [18] were able to measure the recovery of meropenem in each patient using the retrodialysis-by-drug method. This method involves adding a known concentration of the drug to the perfusion fluid being pumped into the catheter (see also section entitled “In vivo recovery experiments” below). After such time that a steady state is reached, the concentration of drug in the microdialysate is measured and compared to the concentration of drug initially added to the perfusion fluid. Dahyot-Fizelier et al. found relative recoveries of 19 ± 7 and 29 ± 7 % for the two patients and calculated brain concentrations of meropenem using these correction factors. Poeppl et al. [19] did not carry out in vivo recovery due to concerns about high concentrations of doripenem in the brain provoking seizures. Instead, they used a value of 38 % recovery that they determined previously, using the microdialysis catheters in soft tissue experiments (unpublished results). The very low apparent brain penetration of doripenem in four out of the five patients in this study might be partly due to low recovery in these particular catheters. However, the authors point out that the flow rate (0.3 μl/min) should be low enough to allow sufficient transfer across the microdialysis membrane.

While in vivo catheter calibration is preferable, it is not always possible, e.g. if the drug is not available in a sterile preparation or if it is otherwise unfeasible to perfuse the drug directly into the brain. Although in vitro microdialysis recovery experiments are only an approximation of how the catheter performs in vivo, they are essential to our understanding of molecular transfer across the microdialysis catheter membrane. Generally, small water-soluble molecules are recovered with high % recovery in laboratory testing, and the lower the flow rate, the higher the % recovery [29–31]. Larger molecules, such as cytokines and chemokines, tend to have lower relative recoveries, depending on physicochemical factors including apparent molecular weight (taking into account dimerization, trimerization, etc.) and isoelectric point (pI) [32].

### Drugs for tumour therapy

Anticancer drugs for treating brain tumours must be able to cross the BBB and accumulate in the tumour tissue at a high enough concentration for a sufficient time to kill tumour cells. Inadequate penetration of the BBB is a significant hurdle for chemotherapy agents in the treatment of cancerous brain tumours [33]. Factors hindering delivery of drugs across the BBB include the multidrug resistance proteins (MRPs) and ATP-dependent efflux pumps such as P-glycoprotein (P-gp), which act as efflux transporters that limit drug access to brain, and to brain tumours. Two recent studies show how microdialysis can be used to assess bioavailability in the development of new anticancer drugs.

Portnow et al. [34] placed microdialysis catheters into residual tumour or peritumoral brain interstitium and measured the concentration of the anti-cancer drug temozolomide after a single oral dose. Temozolomide is a pro-drug that metabolizes in the body to MTIC (3-methyl-(triazen-1-yl)imidazole-4-carboxamide), which is an alkylating agent [35]. It is also a radiosensitizer, and is therefore a key component of chemo-radiation therapy for glioblastomas [36]. The microdialysis catheters were inserted after tumour resection, within 5 mm of the resection cavity. Temozolomide was determined in blood plasma and brain ECF from seven patients using liquid chromatography–tandem mass spectrometry (LC–MS/MS) [34]. The recovery of the drug by microdialysis was measured in vitro, and patients’ results were adjusted accordingly. Retrodialysis recovery determination in vivo for catheter calibration was unfeasible because temozolomide was only available in a non-sterile oral formulation.

Pharmacokinetic parameters for temozolomide in plasma and brain ECF were determined using non-compartmental pharmacokinetic methods [34]. As a measure of brain penetration, the mean brain-to-plasma AUC ratio was 0.178 (range 0.019–0.332). This is similar to the ratio of temozolomide in CSF to plasma reported by Osterman et al. [37], which was 0.2. The time of maximum concentration (T_max_) in the brain ranged from 1.2 to 3.4 h (average 2.0 h), compared with between 0.5 and 4.0 h (average 1.8 h) in the blood. The concentration of drug in brain ECF generally rose more slowly and stayed elevated for longer compared to the drug in plasma (elimination half-life in plasma was 2.1 h average, while the elimination half-life in brain ECF was 2.9 h average).

The authors discuss the importance of their T_max_ measurements in the context of the drug being used as a radiosensitizer before administration of radiation therapy [34]. It is typically given 1 h before radiotherapy, because previous results showed that the drug peaked in plasma 1 h after an oral dose. The authors suggest that this may be suboptimal because the T_max_ in brain is 2 h after oral dose. As it happens, the average T_max_ for plasma in their study is close to 2 h also (1.8 h). Findings from microdialysis may thus be useful for optimising the efficacy of therapies and designing better protocols.

This study also confirms a prediction made by Zhou et al. [38] that the brain-to-plasma AUC ratio would be 0.2 in humans. This prediction was made using microdialysis to measure the pharmacokinetics of temozolomide in rat brain, and subsequent scaling up to predict drug concentrations in human brain.

As discussed in the last section, one of the issues associated with cerebral microdialysis studies on both severely head injured patients and cerebral tumour patients is that there may be BBB impairment in the region of the catheter, leading to increased permeability. This may be useful in getting sufficient concentration of drug into the brain, but could also be damaging if the dose is too high. The extent of BBB impairment can be measured using dynamic contrast-enhanced magnetic resonance imaging (DCE-MRI) [39]. This procedure, which is used for the radiographic assessment of brain tumours, involves taking an MRI scan after intravenous administration of a gadolinium based contrast agent, e.g. gadopentetic acid (Magnevist) and gadobutrol (Gadovist). The contrast agent is confined to the intravascular space until it passes through a region of BBB breakdown where it can permeate into the extravascular space. These regions then show up in the MRI scan as contrast-enhanced compared to normal regions of the brain.

**Fig. 5:**
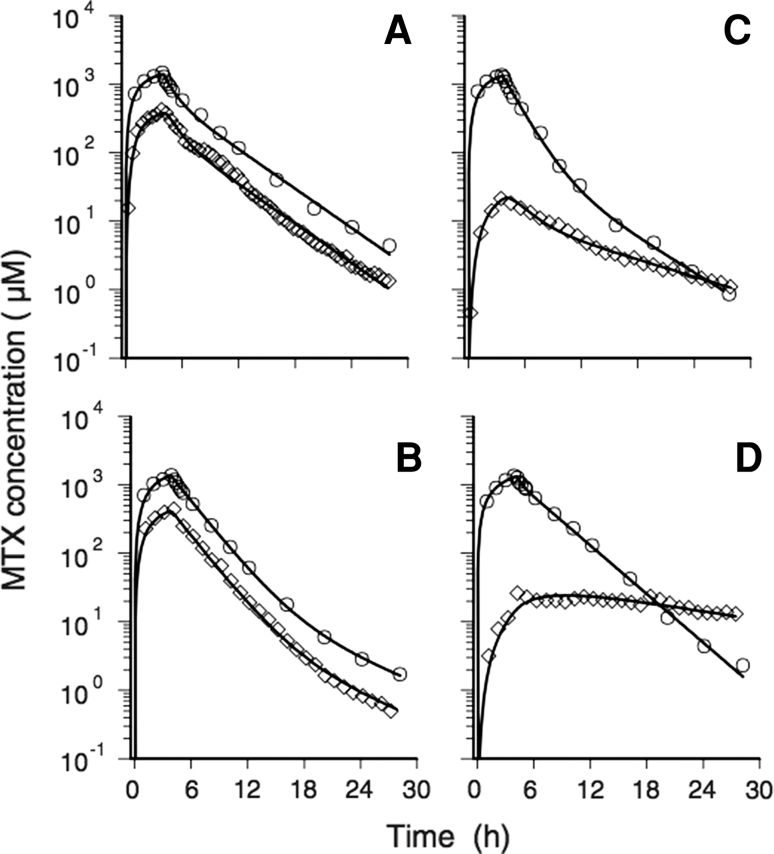
Pharmacokinetics of methotrexate, from a study by Blakeley et al. [40]. **“**Time courses of the MTX concentration in plasma (*white circle*) and brain ECF (*white diamonds*). The plasma profiles are similar in each of the four patients, with peak drug levels ranging from 1,321 to 1,407-μM at the end of the 4-h i.v. infusion of MTX 12 g/m^2^. Time courses of MTX in ECF are dependent upon whether the probe of the microdialysis catheter was placed in contrast enhancing (patients **a** and **b**) or non-enhancing (patients **c** and **d**) regions of the tumor.” Reproduced with kind permission from Springer Science+Business Media: Journal of Neuro-oncology, Effect of blood–brain barrier permeability in recurrent high grade gliomas on the intratumoral pharmacokinetics of methotrexate: a microdialysis study, vol. 91, 2012, pp. 51–58. Blakeley JO, Olson J, Grossman SA, He X, Weingart J, Supko JG, Fig. 2

Blakeley et al. [40] used microdialysis to study the pharmacokinetics of the chemotherapy drug methotrexate in four patients following tumour resection. The authors also used DCE-MRI, in conjunction with computerized tomography (CT) scanning, to report the integrity of the BBB at the exact position of the catheter tip. The microdialysis probe has a gold tip and is visible in the CT scan. Pharmacokinetic analysis showed that the extent of penetration of drug into the brain after infusion depended on the integrity of the BBB in the region of the catheter. If the CT and MRI scans showed that the catheter tip was in a region of BBB disruption, a higher concentration of drug was found: the AUC ratio of brain-to-plasma was 0.281 and 0.305 in these two patients. When the catheter was placed in a contrast non-enhancing region of the tumour, where there was minimal disruption of the BBB, the AUC ratio of brain-to-plasma was much lower, 0.032 and 0.094 for two patients. Another finding was that the rate of decline in brain ECF drug concentration was similar to the rate of decline in plasma drug concentration for the damaged BBB, but elimination from the brain was considerably slower when the BBB was intact (Fig. 5). The authors suggest that there is free exchange of unbound drug molecules between blood and brain ECF in regions where the BBB is disrupted.

Researchers know what concentration of chemotherapy drug is required to kill tumour cells from in vitro work. The average concentration of methotrexate required for 50 % cell kill against various glioma cell lines after 72 h of incubation was reported to be 2.4 μM [41]. Even in the contrast non-enhancing (intact BBB) regions of the tumour, much higher peak concentrations of methotrexate were found. Also, in both sets of patients, the time that brain ECF methotrexate concentration exceeded 2 μM ranged from 20 to 26 h. Thus, the minimum requirement of achieving a potentially cytotoxic concentration within the tumour was attained with methotrexate at the dose used. This small study was thus highly informative, and the authors suggested that phase II trials of chemotherapy drugs should only be carried out if it has first been demonstrated in early clinical studies like this one that therapeutic concentrations in tumour are achievable. Otherwise, without such information, phase II trials may waste valuable resources and patients who could be enrolled into other studies.

Out of the ten patients that were initially recruited for this study, six failed to complete. A high failure-to-complete rate is often one of the pitfalls of small studies like this one. The causes for failure in this study included two patients that were found to have the wrong grade of tumour and two patients that did not receive the drug as specified in the study design. Technical malfunctions accounted for another two failures: a microdialysis catheter membrane ruptured in one case and in another there was a faulty connection between syringe pump and tubing.

### Neuroprotective agents

Microdialysis has been used to study the effects of potentially neuroprotective drugs on neurochemical analytes in patients suffering from TBI.

Cyclosporin A (CsA) is a cyclic peptide (1,203 Da) that is used widely as an immunosuppressant in organ transplantation. It is also being developed as a neuroprotective agent for use in the early phase after TBI because of its ability to preserve mitochondrial activity by inhibiting the opening of the mitochondrial permeability transition pore [42]. Mazzeo et al. [43] used microdialysis to assess the effect of CsA on brain energy metabolism and brain haemodynamics in a randomized, double blind, placebo-controlled study on 50 patients with severe head injury. In addition to monitoring neurochemistry and brain hemodynamics, a second paper reports the clinical safety and tolerability results for the given dose [44]. Also, incidences of adverse events in the first week and overall neurological outcome at 3 and 6 months were reported.

The main finding in the microdialysis study by Mazzeo et al. [43] was that the concentration of glucose in brain ECF was significantly higher in the CsA treated patients than in the patients that received a placebo. This elevation of glucose lasted for at least 2 days after the 24 h infusion of CsA was complete. Both pyruvate and lactate were also higher in the CsA patients. The CsA infusion raised brain extracellular glucose up into the normal range for the entire period of study [45], whereas the brain glucose in patients given placebo was below the normal range for most of this period. This apparent normalisation of brain glucose appeared independent of plasma glucose, so was attributed to a cerebral mechanism. The significantly higher microdialysate lactate in the CsA patients compared to those given placebo appeared to refute the original hypothesis that CsA would improve mitochondrial function. Lactate is a product of glycolysis and its increase is indicative of mitochondrial dysfunction or (if relevant) lack of oxygen. However, lactate is also a fuel for neurons, so lactate increase *per se* is not necessarily deleterious, especially as microdialysate pyruvate concentration also increased, suggesting some preservation of mitochondrial function, in the CsA group compared with placebo. An important indicator is the microdialysate lactate/pyruvate (L/P) ratio, which was similar in both CsA and placebo groups during the 24 h of infusion, while at 3 and 4 days post-infusion the L/P ratio was significantly lower in the CsA group, suggesting an improvement in energy metabolism.

The mean peak concentration of CsA in CSF was 2.08 ng/ml during the 24-h infusion of the drug [43]. CsA concentrations detected in microdialysates ranged from zero to 0.61 ng/ml, without adjustment for relative recovery (G.M. Brophy—personal communication). Both the CSF and microdialysis data suggested very limited brain uptake, in view of the much higher mean steady-state concentration of CsA in whole blood, which was 545 ng/ml during the infusion. However, the in vitro recovery of CsA using the microdialysis catheters is also very low. Due to its lipophilic nature, CsA binds non-specifically to glass and plastic (PVC) surfaces [46], and probably to the microdialysis membrane. So, although the administration of CsA causes a significant change in brain chemistry, the concentration of drug in the brain ECF required to achieve the effect is still not known.

In the second paper looking at the safety and tolerability of CsA, Mazzeo et al. [44] assess the effect of CsA on renal function, hepatic function, blood cell parameters, occurrence of adverse events and neurological outcome at 3 and 6 months. They found no difference in neurological outcome between the CsA patients and the placebo patients, suggesting that the elevated glucose concentrations in the brain had no effect on outcome.

In contrast, a study by Hatton et al. [47] showed a dose-related improvement in favourable outcome when patients were given CsA within 8 h of injury. These authors suggest that TBI may temporarily alter the BBB permeability and there may be a window of dosing opportunity when CsA can penetrate the BBB. The authors speculate that if the CsA is administered within 8 h, the drug can find its way into the brain where it can have its neuroprotective effects. Sullivan et al. [48] suggest the reason why Mazzeo’s study [44] failed to show an outcome was that CsA was administered after the 8-h window. Of course, the only way to prove this would be to accurately determine the concentration of CsA in brain ECF.

### Anticonvulsant drugs

One of the challenges in treating epilepsy is that up to 30 % of patients continue to have seizures even when they are receiving anti-epileptic drug treatments, i.e. refractory epilepsy. The causes are not known, but inadequate drug concentration in crucial brain areas is a possible contributing factor. One reason for this may be over-expression of efflux transporters in patients with epilepsy in response to administration of antiepileptic drugs, as was found for phenytoin [49].

**Fig. 6:**
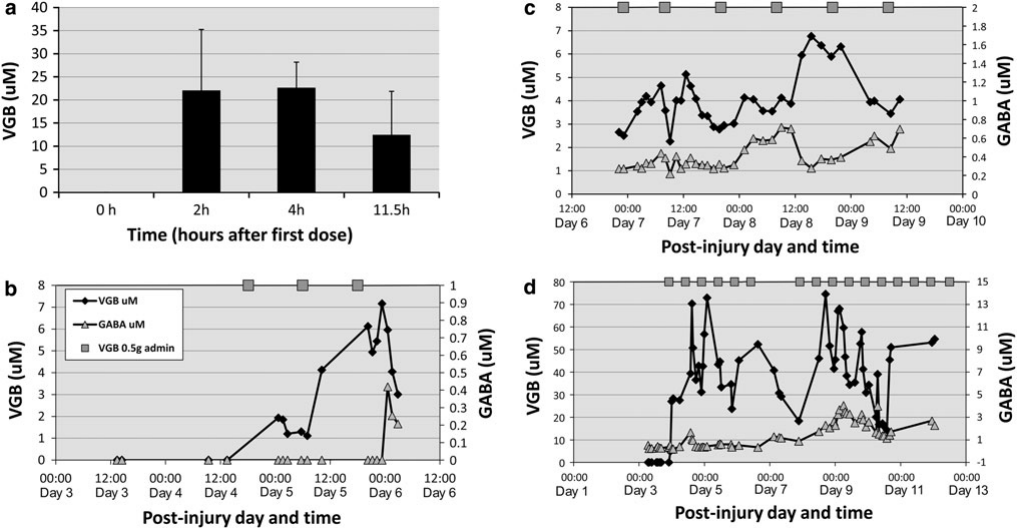
Levels of vigabatrin (VGB) and GABA in TBI patients versus time, adapted from Carpenter et al. [50]. VGB was administered enterally (0.5 g twice daily). **a** Concentrations (mean ± SD) of VGB (μmol/L) in blood plasma for the first half-day following the first VGB dose, for five patients. **b** Concentrations of VGB (μmol/L) (indicated by *black diamonds*) and GABA (μmol/L) (*grey triangles*) versus time in brain microdialysates from patient W (male, 17 years, admission GCS 3). Day 0 is the day of injury. Microdialysis started on day 3 post-injury, and VGB treatment commenced on day 4 (18:00). Data are for the first 1.5 days of VGB administration. The times of VGB doses are indicated by *grey squares*. **c** Concentrations of VGB (μmol/L) and GABA (μmol/L) in brain microdialysates from patient R (male, 33 years, admission GCS 14) versus time, for the first 2.5 days of VBG administration. Microdialysis commenced on day 1 post-injury, and VGB treatment commenced on day 6 (23:05). Symbols are as in *panel* (**b**). **d** Concentrations of VGB (μmol/L) and GABA (μmol/L) versus time in brain microdialysates from patient C (male, 66 years, admission GCS 7) for the first 8.5 days of VGB administration. Symbols are as in *panel* (**b**). Microdialysis commenced on day 2 post-injury, and VGB treatment commenced on day 3 (22:00). There was a gap of 36 h between the sixth and seventh doses

An anticonvulsant drug of interest is vigabatrin (gamma-vinyl-gamma-aminobutyric acid), an inhibitor of GABA-transaminase (GABA-T), an enzyme responsible for degrading GABA. Vigabatrin is thus regarded as neuroprotective, and has been studied by cerebral microdialysis in TBI patients (0.5 g every 12 h, enterally) with multimodality monitoring and a preliminary report published [50]. Vigabatrin is a small water-soluble molecule (129 Da) and, as expected, is efficiently recovered by microdialysis (in vitro recovery 100 %, R.J. Shannon unpublished observation). Microdialysis results from three patients are illustrated in Fig. 6, showing that vigabatrin levels rose in brain microdialysates, followed by modest increases in GABA [50]. Vigabatrin and GABA levels increased more in abnormal brain (patient C, Fig. 6d) than in sites further from lesions (patients W and R, Fig. 6b, c), and were higher after multiple vigabatrin doses than after one dose. Highest vigabatrin and GABA levels were 75 and 4 μM respectively. Vigabatrin did not overtly affect intracranial pressure and other pressure parameters, or microdialysate lactate, pyruvate and L/P ratio in this preliminary evaluation [50]. This study has demonstrated (albeit in a small number of patients) the principle that multimodality monitoring, including cerebral microdialysis, is feasible for studying surrogate end-points following administration of a putative neuroprotective drug.

## Challenges and solutions

### Limitation of patient numbers

Human cerebral microdialysis is an invasive technique, which limits its applicability because the catheter must be surgically implanted into the brain. Examples of eligible patients are those requiring surgery for brain tumour resection or requiring treatment for TBI in NCCUs. There are a very limited number of these patients available to study, so only small observational studies or limited clinical trials are feasible.

### Variability of microdialysis results

The patients recruited into cerebral microdialysis studies usually have traumatic or non-traumatic brain injuries or tumours. Results obtained from a study on such patients might not necessarily extrapolate to the kind of patient most likely to benefit from the drug being studied. Also, as illustrated in this review, there can be significant variation in the brain permeability of a drug between patients in the study. This is because brain injuries or tumour growth can cause BBB disruption, which can be slight or severe, localised or general. The position of the microdialysis catheter with respect to brain injury or tumour is therefore an additional variable in these studies.

### Microdialysis recovery of drug from the brain ECF

There are several reasons why a drug may be poorly recovered from brain ECF by microdialysis. Lipophilic molecules have been shown to bind non-specifically to various parts of the microdialysis catheter (e.g. membrane and/or tubing) [51]. Researchers have included additives in the microdialysis perfusion fluid, including albumin [52] and β-cyclodextrin [53], in attempts to improve the recovery of lipophilic molecules.

Non-recovery of a drug from the brain ECF may be simply because it is not there. Unless the drug is small and lipophilic, it can only enter the brain if it is a substrate for a BBB transporter or if the BBB is disrupted [5]. Even if a drug does enter the brain, it may be efficiently exported back across the BBB by efflux transporters, e.g. P-gp and/or MRPs. This is the brain’s self-defence mechanism that has presumably evolved naturally as a protection against harmful substances, and which often works against the development of new neuroactive drugs. Another reason for a drug not being detected in the brain ECF is that it may be rapidly taken up by cells, or become bound to extracellular matrix, or be extensively metabolised by cells.

### In vivo recovery experiments

Several techniques exist for calculating relative recovery in vivo. One of these is the extrapolation-to-zero-flow technique [29] [2]. This involves varying the flow rate through the microdialysis catheter. As the flow rate falls, recovery will rise as the microdialysate has more time to equilibrate with the ECF. The logarithm of the concentration of the substance of interest (y-axis) is plotted against flow rate (x-axis) and a line fitted to the data points. Extrapolating this fitted line to zero flow (i.e. determining the intercept on the y-axis) gives the “true” ECF concentration of the substance, and enables calculation of relative recovery at each of the flow rates previously employed. This technique has the disadvantages that it is time-consuming and relies on the concentration of the substance of interest remaining constant in the ECF while the flow rate variations are being carried out. Such constancy cannot be assumed in a complex biological environment, and so a reference catheter is employed at a constant flow rate in nearby tissue to detect fluctuations and enable the concentrations to be corrected for the test catheter, a procedure that in turn relies on certain assumptions. The extrapolation-to-zero flow method is not practicable in pharmacokinetic studies.

Another in vivo recovery determination method is retrodialysis-by-drug, as used by Dahyot-Fizelier et al. [18] in their meropenem study (see above). This involves perfusing the microdialysis catheter with a known concentration (C_in_) of drug until the emerging concentration (C_out_) of drug in the microdialysate is steady. Relative recovery is calculated from the concentration difference expressed as a percentage, i.e. relative recovery = 100 × (C_in_ − C_out_)/C_in_. Retrodialysis by calibrator, i.e. a substance chosen as being closely related to the drug, is another variant of the method [54]. Retrodialysis-by-drug is widely used in pharmacokinetic studies, but it is unsuitable for determining relative recoveries of endogenous molecules.

A further in vivo recovery determination method is the no-net-flux method [2, 55]. The concentration of the substance of interest, e.g. drug, is varied in the perfusate. If the concentration of drug in the perfusate (C_in_) is greater than in the surrounding ECF then the substance will diffuse out of the perfusate into the ECF, so the emerging concentration of drug in the microdialysate (C_out_) is lower than C_in_. Conversely, if the ECF concentration of the drug is higher in the ECF than in the perfusate, then the drug will diffuse out of the ECF into the perfusate, so the emerging microdialysate C_out_ will be higher than C_in_. A plot of the difference between C_in_ and C_out_ (C_diff_, y-axis) versus C_in_ (x-axis) is then constructed, and a line is fitted. The concentration at which C_diff_ is zero (i.e. where the line crosses the x-axis) is the “true” concentration of the substance in the ECF. While this method is often regarded as the “gold standard” and is suitable for both endogenous and exogenous substances, it has the disadvantage that it needs to be done at a steady state and is not suitable under transient conditions.

In vivo recovery experiments such as retrodialysis are not always feasible. For example, when the drug is not available in a formulation suitable for retrodialysis or when direct perfusion of drug into the brain may cause adverse events such as seizures. In these cases, the recovery rates can only be estimated e.g. from in vitro bench tests. Even so, useful information can still be obtained from patients by comparing changes in concentrations over time, e.g. before and after dose, and accompanying changes in any other relevant analytes (e.g. glucose, lactate, pyruvate etc.) and/or downstream target molecules of the drug.

### Regular and continuous sampling

One of the advantages of microdialysis is that it is a continuous-flow technique. In the NCCU, microdialysis collection vials are routinely changed every hour, enabling detailed time-course measurement of drug concentration. However, because the care of the patient is the primary concern, microdialysis sampling may not always be optimal for the purposes of drug pharmacokinetic studies. For example, the patient may be moved for a scan or operation and the microdialysis pump temporarily disconnected for the duration. Other essential procedures on the ward may mean that vial change times are disrupted. Also, occasionally there can be malfunctions in microdialysis pumps and/or catheters. Such irregularities can pose problems for pharmacokinetic studies.

### Regional distribution in brain

A drug may produce its therapeutic effect in one region of the brain, but actually accumulate in a different region of the brain. Kornhuber et al. [56] have reported region-specific distribution of the neuroleptic drug levomepromazine in post-mortem brain tissue. The authors looked at brain tissue samples from 15 subjects who had been treated orally with either levomepromazine or haloperidol, both neuroleptic drugs. They found that levomepromazine distributed unevenly, with a significant difference between the cortex and the basal ganglia regions. The analysis of brain tissue does not distinguish between unbound drug and cell-associated drug. However, this study does illustrate that a drug may accumulate in one region in preference to another, which is important to bear in mind in microdialysis studies of patients. Microdialysis is a very focal technique, so the microdialysis catheter needs to be placed in a relevant, clinically valid site. Although in some patients it is possible to insert two microdialysis catheters, to compare different sites, microdialysis cannot provide broad regional or global information. Combined microdialysis and scanning studies may be more relevant in this context.

### Genetic polymorphism and BBB transporters in patients

A considerable amount is known about BBB transporters and their substrates (for example, see Taylor et al. [57]). Of these, the best characterised is P-gp, also termed multidrug resistance protein or MDR-1, which is a member of the ATP-binding superfamily [59, 60]. P-gp is ubiquitous at many endo- and epithelial barriers, not least the BBB, and may act as an efflux transporter for up to 50 % of clinically important drug compounds. Correspondingly, P-gp knockout animals can show 10- to 100-fold increases in brain concentrations of drugs that are substrates for this transporter [58].

Certain genetic polymorphisms in P-gp have emerged as potentially important in determining the efficacy and incidence of adverse effects of specific neuroactive drugs. For example, two single nucleotide polymorphisms in P-gp have been associated with clinically significant increase in side effects of the dopamine agonist cabergoline [59]. In contrast, Brunner et al. [60] carried out a PET study to find out if patients with P-gp variants had altered penetration of the labelled molecule ^11^C-verapamil and found no difference between the two groups above inter-individual variability, concluding that genetic variants of P-gp had no significant effect on BBB penetration of this specific drug. Overall, it is still largely unclear how to predict the magnitude of effect that common P-gp polymorphisms will have on CNS bioavailability for any individual drug [12]. Consequently, careful consideration should be given to genetic profiling of patients in microdialysis pharmacokinetic studies as polymorphisms in efflux transporters may be an important source of observed variability in CNS drug concentrations.

### Measuring the integrity of the blood brain barrier

Transport of drugs across the BBB can be passive or active. Passive transport increases if the integrity of BBB is compromised. The BBB is dynamically controlled by components in the blood and in brain ECF. For instance, inflammatory mediators released from astrocytes may affect transport across the BBB. Dynamic regulation of the BBB means that its behaviour may vary in different situations.

Beyond assessing the penetration of individual drugs into the CNS, microdialysis could be employed to measure the general integrity of the BBB. In diverse pathological conditions the apical tight junctions between endothelial cells that inhibit passive diffusion are disrupted through proteolysis and down-regulation of their component proteins. Key mediators commonly implicated in this process are vascular endothelial growth factor (VEGF), matrix metalloproteinases (MMPs, particularly −2 and −9), and nitric oxide [61–63]. Where the BBB is no longer effective there is potential to develop vasogenic oedema and swelling with resulting brain shift and raised intracranial pressure, both of which have adverse implications on patient outcomes.

The degree of BBB impairment is usually quantified by measuring interstitial brain concentrations of a tracer compound to which the BBB is normally impermeable following bolus intravascular injection. Tracers with a range of molecular weights can be employed to gauge the severity of BBB disruption. Common low molecular weight tracers include radiolabelled sucrose or fluorophores such as fluorescein; larger molecular weight tracers include fluorescent or radiolabelled albumin and dextrans [64, 65]. In animal studies the extent of extravasation is typically assessed by autoradiography or microscopy of brain slices, or assayed directly in tissue homogenate. In humans, non-invasive radiological techniques to measure BBB permeability utilise dynamic computed tomography or magnetic resonance imaging (MRI) following intravenous injection of iodinated or paramagnetic contrast agents, respectively [66, 67]. In the microdialysis study described in section entitled “Drugs for tumour therapy”, Blakeley et al. [40] used dynamic contrast enhanced MRI to measure BBB permeability. Alternatively, there are also SPECT and PET tracers, usually radiolabelled EDTA or DTPA, which have been synthesised for measuring BBB permeability in vivo [70, 71].

Microdialysis has the potential to be used for in vivo monitoring of BBB permeability using a similar paradigm, with several potential advantages. The ideal tracers would be endogenous plasma molecules that do not normally cross the BBB (e.g. albumin) [68], although serial injection of exogenous tracer would also be feasible [69]. The principal benefits of a microdialysis approach to measuring BBB integrity would be continuous, potentially on-line, in vivo assay of multiple tracers of differing molecular weights, as opposed to one-time ‘snapshots’ of permeability to a single contrast agent provided by CT or MRI. Nonetheless, microdialysis cannot provide the spatial coverage of CT or MRI and these techniques should be viewed as complementary.

This application of microdialysis would be particularly useful for determining the efficacy of therapeutic strategies targeted at reducing BBB permeability and brain oedema in conditions such as TBI.

Dynamic contrast-enhanced MRI has been used to detect subtle changes in the BBB [70], and while other researchers have highlighted the need for further technical development [71], the technique has potential for studying TBI patients. Other BBB measurement methods exist, such as perfusion CT with iodinated contrast agents [66] (see above). However it should be borne in mind that BBB permeability is not a simple phenomenon and will vary depending on the molecular species in question.

### Comparing extracellular and intracellular concentrations

Microdialysis measures extracellular concentrations, and cannot measure intracellular concentrations of molecules. However, microdialysis can be usefully employed to complement scanning techniques that measure total tissue concentrations. For example, Langer et al. [72] studied the intracellular drug pharmacokinetics of ^18^F-ciprofloxacin using a combined microdialysis and PET study. Although this study was in human muscle, it illustrates a principle that could potentially be applied in human brain, and such studies have been carried out in animals [73]. PET measures total radiolabelled drug concentration (extracellular, intracellular and intravascular), and microdialysis measures extracellular concentration. Therefore the difference gives a method to measure the pharmacokinetics of the drug intracellular concentration.

## Conclusions and future prospects

Cerebral microdialysis is proving its worth as a clinical monitoring technique for severe brain injury, in which fundamental molecules, such as glucose, lactate and pyruvate, are measured at the bedside [74, 75], and is increasing its applicability to the study of drugs in the human brain as exemplified in the present review. As well as microdialysis providing drug concentration data in the brain ECF, the ability to simultaneously measure other biomarkers within the microdialysate may provide a method for assessing the downstream effects of pharmacological agents. For example, microdialysis can reveal valuable information on whether putative neuroprotective agents can improve brain chemistry in terms of L/P ratio, glucose etc.

Microdialysis is the only existing technique for sampling molecules from the brain ECF. As an invasive technology, it is limited to certain categories of patient—those requiring neurocritical care and/or neurosurgery—but this is not necessarily such a drawback in itself as such patients are likely to benefit from development of CNS drug therapies, and extrapolation to related patient groups seems possible, such as certain forms of epilepsy. However, there is a much larger and well-recognised need for better CNS drugs to treat chronic neuro-pathologies that appear more widespread in the general population, such as multiple sclerosis and Alzheimer’s disease. Such patients are currently inappropriate for the degree of invasiveness inherent in cerebral microdialysis. For the present, cerebral microdialysis in neurocritical care and/or neurosurgery patients can provide surrogate information that may be relevant in general terms of neuroprotection strategies. Small phase II clinical trials utilising cerebral microdialysis would seem crucial precursors to deciding on which drugs are promising enough to progress to larger, more costly trials. Cerebral microdialysis may also pave the way for future technology development. In the longer term, sensor devices that are smaller and less invasive than the existing microdialysis catheters may be developed for implantation into brain, and open up new prospects for CNS drug development.
